# SNF1-Related Protein Kinase 1 Activity Represses the Canonical Translational Machinery

**DOI:** 10.3390/plants11101359

**Published:** 2022-05-20

**Authors:** Seungmin Son, Jong Hee Im, Giha Song, Sang Ryeol Park

**Affiliations:** 1 National Institute of Agricultural Sciences, Rural Development Administration, Jeonju 54874, Korea; linewind@korea.kr (S.S.); jhim@msu.edu (J.H.I.); geometry@korea.kr (G.S.); 2 Department of Life Sciences, Korea University, 145 Anamro Seungbukgu, Seoul 02841, Korea; 3 Department of Horticulture, Michigan State University, East Lansing, MI 48824, USA

**Keywords:** Arabidopsis, mRNA translation, polysome, rice, SNF1-related protein kinase 1

## Abstract

Protein biosynthesis is achieved through translation, which consumes enormous energy. Therefore, under conditions of limited energy supply, translation progress should be strictly coordinated. Sucrose non-fermenting kinase1 (SNF1)-related protein kinase 1 (SnRK1) is an evolutionarily conserved master regulator of cellular energy stress signaling in plants. Rice (*Oryza sativa*) and Arabidopsis (*Arabidopsis thaliana*) SnRK1 enhance hypoxia tolerance and induce the expression of stress-related genes. However, whether SnRK1 modulates protein synthesis in plants is unknown. In this study, using translational reporter constructs transfected in Arabidopsis protoplasts we showed that the expression of *OsSnRK1A* and *AtSnRK1.1* decreases the abundance of canonical proteins without affecting their encoding transcript levels and protein stability. Moreover, the loading of total mRNAs and *GFP* mRNAs into the heavy polysome fraction which is normally translated was attenuated in transgenic Arabidopsis lines constitutively expressing *OsSnRK1A* or *AtSnRK1.1*. Taken together, these results suggest that OsSnRK1A and AtSnRK1.1 suppress protein translation to maintain energy homeostasis.

## 1. Introduction

Energy starvation caused by the removal or absence of an energy source is a major threat to the survival of all living organisms [1]. Under such energy stress conditions, anabolic processes that consume energy are inhibited and catabolic processes that release energy are promoted at the cellular level to maintain energy homeostasis [2]. The translation of protein from messenger RNA (mRNA) is an enormously energy-consuming process [3]. Therefore, under energy stress conditions such as those caused by glucose deficiency or hypoxia, the translation involved in canonical protein synthesis is strongly suppressed to adjust to an environment with low adenosine triphosphate (ATP) availability [4,5].

The 5′-adenosine monophosphate (AMP)-activated protein kinase (AMPK) is a serine/threonine protein kinase that acts as the central regulator of energy stress signaling [6,7]. AMPK is a highly conserved trimeric protein complex that detects the ratio of AMP to ATP [8]. AMPK is activated by energy deficiency and regulates various signaling and metabolic pathways to maintain energy homeostasis in eukaryotic cells [9]. In particular, AMPK suppresses cap-dependent protein translation at multiple levels to conserve energy while promoting the cap-independent and internal ribosome entry site (IRES)-dependent translation for transcripts required for cell survival under energy crisis [5,10].

In plants, sucrose non-fermenting kinase1 (SNF1)-related protein kinase 1 (SnRK1), the ortholog of animal AMPK, is an evolutionarily conserved energy sensor [11,12]. SnRK1 is a heterotrimeric enzyme composed of one catalytic α subunit and two β and γ regulatory subunits [13]. Activation of SnRK1 modulates the transcriptome reprogramming to adapt plants to stress conditions [14]. Besides transcriptional controls, SnRK1 promotes catabolism and represses anabolism in plants. SnRK1 phosphorylated the autophagy-related gene 1 (ATG1) and enhanced the autophagy required to maintain homeostasis in nutrient deprivations [15]. This plant energy stress master regulator is also involved in the translation process. Under energy stress conditions due to hypoxia, the initiation of translation drops globally in Arabidopsis (*Arabidopsis thaliana*) seedlings while the abundance of transcripts for hypoxia-induced genes increases in polysome complexes [16]. Furthermore, SnRK1 phosphorylates the eukaryotic translation initiation factor (eIF) isoform 4G1 (eIFiso4G1), resulting in the higher translational efficiency of specific transcripts, including those induced by hypoxia during submergence [17]. However, phosphorylation of the cap binding proteins such as eIF4E and eIFiso4E by SnRK1 led to a global translation inhibition [18]. Therefore, the most striking effect of SnRK1 seems to be the inhibition of global translation in plants [19]. Nevertheless, SnRK1-mediated translational regulation in plants is poorly understood, even though crop SnRK1s are involved.

Previous studies have shown that rice (*Oryza sativa*) SnRK1 (OsSnRK1A) and Arabidopsis SnRK1.1 (AtSnRK1.1) regulate the expression of flooding response genes and enhance stress tolerance against energy deficiency conditions due to submergence in Arabidopsis [20,21]. However, whether SnRK1 activity regulates the translational machinery is unclear. In this study, we demonstrate through a combination of in vitro methods, protoplast transfection assays, and transgenic plants that OsSnRK1A and AtSnRK1.1 inhibit canonical protein synthesis in Arabidopsis.

## 2. Results

**Figure 1:**
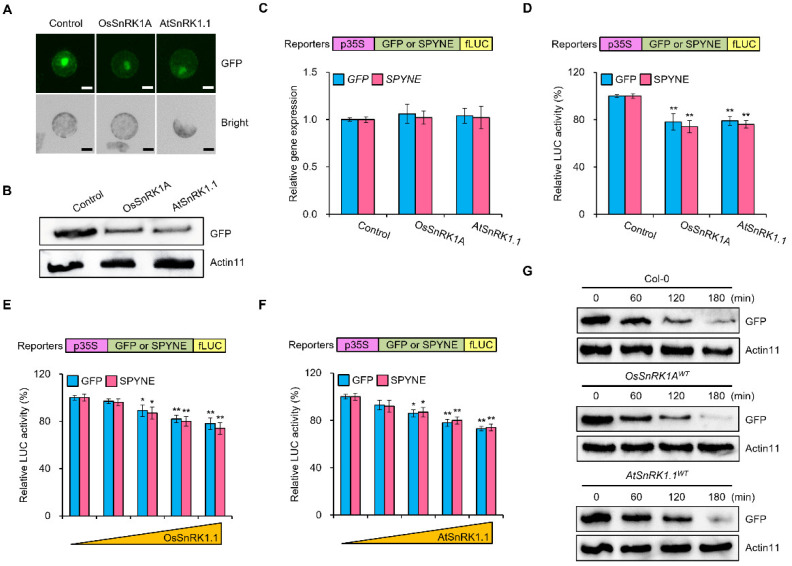
OsSnRK1A and AtSnRK1.1 repress canonical protein biosynthesis. (**A**,**B**) GFP accumulation in protoplasts. *GFP* was transfected into protoplasts alone or together with *OsSnRK1A* or *AtSnRK1.1*. GFP signals after a 6-h incubation were observed by fluorescence microscopy (**A**), and GFP abundance determined by immunoblot with an anti-GFP antibody (**B**). Scale bar: 10 μm. Actin11 was used as a loading control. (**C**–**F**) Transcript levels and activities of the reporters for translated proteins. The reporter genes (*GFP* and *SPYNE*) were transfected into protoplasts alone or together with *OsSnRK1A* or *AtSnRK1.1*. Samples were collected after a 6-h incubation in WI solution. Relative transcript levels of the reporters were analyzed by RT-qPCR using *AtACTIN* as an internal reference (**C**). Relative activities of the reporters were measured by a luciferase system, with *proUBQ10-rLUC* serving as an internal reference (**D**). Activity of the reporters for translated proteins with different amounts of co-transfected construct expressing *OsSnRK1A* (**E**) or *AtSnRK1.1* (**F**), respectively. Data are shown as means ± standard deviation (SD). Asterisks indicate statistically significant differences relative to controls (* *p* < 0.05, ** *p* < 0.01). (**G**) Cell-free GFP degradation assay. Purified GFP proteins were incubated with total protein extracts from Col-0, *OsSnRK1A^W^^T^*, or *AtSnRK1.1^W^^T^* for the indicated times. Immunoblots were probed with anti-GFP antibody. Actin11 served as a loading control.

To determine whether plant SnRK1s regulate canonical protein synthesis, we transfected Arabidopsis leaf mesophyll protoplasts with constructs that were overexpressing green fluorescent protein (*GFP*) alone or together with *OsSnRK1A* (Os05g45420) or *AtSnRK1.1* (At3g01090). We then observed the GFP fluorescence of these protoplasts after a 6-h incubation in washing and incubation (WI) solution. We detected much weaker GFP fluorescence signals in the protoplasts expressing *OsSnRK1A* or *AtSnRK1.1* compared to protoplasts transfected with *GFP* alone (Figure 1A). To confirm these results, we assessed the GFP abundance by immunoblot assay which confirmed the lower abundance of GFP upon expression of *OsSnRK1A* or *AtSnRK1.1* (Figure 1B). To obtain more quantitative results, we designed reporters for protein translation using GFP or SPYNE (the N terminus of split yellow fluorescent protein [YFP]) as exemplar proteins synthesized via canonical translation. We then transfected each construct alone or together with *OsSnRK1A* or *AtSnRK1.1*, respectively (Figure 1C,D). To distinguish between the transcriptional and post-transcriptional effects of SnRK1 on the reporters we measured relative *GFP* and *SPYNE* transcript levels by reverse transcription quantitative polymerase chain reaction (RT-qPCR). The co-transfection of the reporter constructs with *OsSnRK1A* or *AtSnRK1.1* did not affect the transcript levels of the translational reporters (Figure 1C). By contrast, firefly luciferase (fLUC) activity, used here as a proxy for the translation potential of each reporter construct, decreased by about 25% when *OsSnRK1A* or *AtSnRK1.1* was co-transfected into protoplasts (Figure 1D). Importantly, fLUC activity derived from the translated protein reporters decreased in a dose-dependent manner with increasing amounts of *OsSnRK1A* construct DNA (Figure 1E). We obtained similar results with increasing amounts of the *AtSnRK1.1* construct (Figure 1F).

Since fLUC activity from the translated protein reporters decreased in the presence of SnRK1s, while their transcription levels did not appear to change (Figure 1C,D), we examined whether the kinases induced protein degradation. To this end, we turned to cell-free degradation assays in which GFP was produced in the protoplasts transfected with a construct overexpressing *GFP* and isolated with GFP-Trap beads. We then incubated the purified GFP with the total proteins extracted from Col-0 seedlings, or seedlings stably overexpressing *OsSnRK1A^WT^* or *AtSnRK1.1^WT^*, and measured GFP abundance by immunoblot. We observed a similar gradual decrease in the abundance of purified GFP over time in all samples (Figure 1G). This result suggested that OsSnRK1A and AtSnRK1.1 do not promote the degradation of GFP.

**Figure 2:**
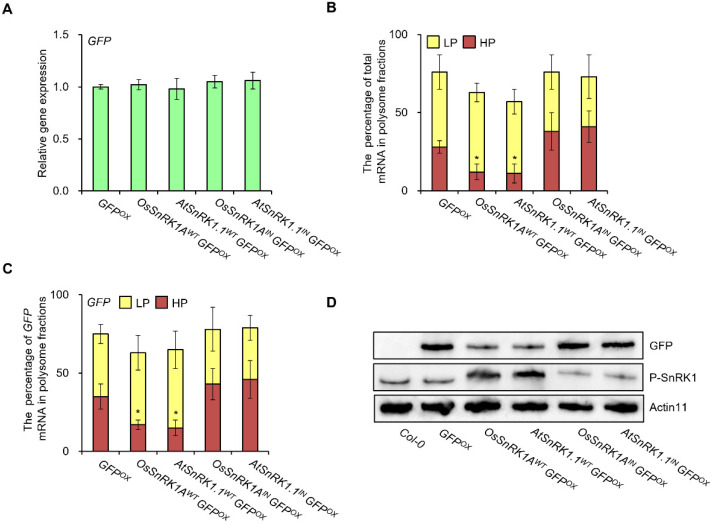
OsSnRK1A and AtSnRK1.1 activity decreases the canonical protein translation. (**A**) Relative *GFP* transcript levels in the indicated transgenic lines. Total RNAs were extracted from 10-day-old seedlings, and the expression levels were measured by RT-qPCR with *AtACTIN* as an internal reference. Data are shown as means ± SD. (**B**,**C**) Percentage of mRNA loaded onto polysomes. Total RNA and polysomes were isolated from 10-day-old seedlings of *GFP^OX^*, *OsSnRK1A^WT^ GFP^OX^*, *AtSnRK1.1^WT^ GFP^OX^*, *OsSnRK1A^IN^ GFP^OX^*, and *AtSnRK1.1^IN^ GFP^OX^*. The total amount of mRNA in the polysome-free (NP), light polysome (LP), and heavy polysome (HP) fractions together was set to 100% and their individual percentages of mRNA were determined by dividing the amount of mRNA in each fraction by the total amount of mRNA (**B**). The amount of *GFP* mRNA in each polysome fraction was measured by RT-qPCR with *DAP1* as an internal reference and expressed as a percentage of the total amount in all three fractions (**C**). Data are shown as means ± SD. Asterisks indicate statistically significant differences from controls (* *p* < 0.05). (**D**) GFP abundance in 10-day-old seedlings of *GFP^OX^*, *OsSnRK1A^WT^ GFP^OX^*, *AtSnRK1.1^WT^ GFP^OX^*, *OsSnRK1A^IN^ GFP^OX^*, and *AtSnRK1.1^IN^ GFP^OX^*. Blots were probed with anti-GFP and anti-pT172-AMPKα antibodies. The anti-pT172-AMPKα antibody recognizes T-loop phosphorylated SnRK1.1 (P-SnRK1.1) reflecting the activated form of SnRK1.1. Actin11 was used as a loading control.

Given that OsSnRK1A and AtSnRK1.1 did not affect transcription or protein stability, we investigated whether they might repress mRNA translation. Accordingly, we generated double transgenic lines by introducing a transgene overexpressing *GFP* into stable transgenic lines overexpressing wild-type *SnRK1* (*OsSnRK1A^WT^* and *AtSnRK1^WT^*) or a SnRK1 variant with a mutation in the ATP-binding site (*OsSnRK1A^IN^* and *AtSnRK1^IN^*). We selected double homozygous T_3_ transgenic lines expressing *GFP* at levels similar to the transgenic plant expressing *GFP* (*GFP^OX^*) for further analyses (Figure 2A) and measured the abundance of total and *GFP* mRNAs in polysome-free (NP), light polysome (LP), and heavy polysome (HP) fractions. We discovered that total mRNAs, which were abundant in the heavy polysome fraction in *GFP^OX^*, accumulated to only 40–50% of *GFP^OX^* levels in the HP fraction in *AtSnRK1.1^WT^ GFP^OX^* and *OsSnRK1A^WT^ GFP^OX^* lines (Figure 2B). To confirm that SnRK1s mediate the drop in GFP abundance due to diminished translation we quantified *GFP* mRNA in the polysome fractions. We determined that the abundance of *GFP* mRNA also decreased in the HP fraction to a similar extent as the total mRNAs in *OsSnRK1A^WT^ GFP^OX^* and *AtSnRK1.1^WT^ GFP^OX^* lines compared to *GFP^OX^* (Figure 2C). Since translated mRNAs are associated with heavy polysomes (HP) [22], these results indicated that OsSnRK1A and AtSnRK1.1 activity represses the translation of canonical proteins, for which GFP was used here as a proxy. Finally, the above results suggested that GFP abundance would be lower in some samples which we tested by immunoblot analysis using the total protein extracts from all transgenic plants. Indeed, we detected lower levels of GFP in *OsSnRK1A.1^WT^ GFP^OX^* and *AtSnRK1.1^WT^ GFP^OX^* lines compared to *GFP^OX^* (Figure 2D). Taken together, these results suggest that the activity of OsSnRK1A and AtSnRK1.1 represses canonical protein synthesis by modulating translational progression.

## 3. Discussion

Plants are photoautotrophic organisms that convert light energy, water, and carbon dioxide into oxygen and chemical energy. Therefore, stress conditions such as hypoxia and flooding attenuate photosynthetic output and are one of the primary energy threats to plants. Under such energy deficiency conditions, plants must rebalance growth/development and metabolism to maintain energy homeostasis [23]. Activation of SnRK1, a key sensor of energy stress signaling, results in convergent reprogramming of the transcriptome and global metabolism to adapt to and survive such cellular energy crises [24,25]. In mammalian cells, AMPK-mediated translational inhibition and its regulatory mechanism have been reported at multiple steps [26]. However, the SnRK1-mediated translational regulation mechanism remains to be elucidated largely in plants.

Rice and Arabidopsis SnRK1s play critical roles as central regulators of flooding stress that is responsible for cellular energy deficiency [20,21]. In this study, we investigated whether the activity of OsSnRK1A and AtSnRK1.1 might regulate protein synthesis and revealed that they in fact suppress mRNA translational progression in Arabidopsis. A previous report showed that AtSnRK1.1 enhances specific protein synthesis, using transgenic plants accumulating a dominant-negative form of AtSnRK1.1 [17]. Here, we raised SnRK1 activity by overexpression of the wild-type form and observed the consequences on mRNA translation progress. The information gathered here will contribute to future studies related to the different roles of SnRK1 in protein translation for canonical and specific mRNAs at multiple steps.

## 4. Materials and Methods

### 4.1. Plant Materials and Growth Conditions

Seeds of the *Arabidopsis thaliana* accession Columbia-0 (Col-0) were germinated and grown on full-strength Murashige and Skoog (MS) medium or on soil under a 16-h light/8-h dark photoperiod at 23 °C. The transgenic plants *OsSnRK1A^WT^*, *AtSnRK1.1^WT^*, *OsSnRK1A^IN^*, and *AtSnRK1.1^IN^* were previously generated and confirmed [20,21]. Transgenic plants overexpressing *GFP* (*GFP^OX^*) were generated using Agrobacterium (*Agrobacterium tumefaciens*) strain GV3101 harboring the pCAMBIA1302 vector by the floral dip method [27]. Homozygous T_3_ lines were selected for assays. F_1_ hybrid plants were generated by crossing *OsSnRK1A^WT^*, *AtSnRK1^WT^*, *OsSnRK1A^IN^*, and *AtSnRK1^IN^* with *GFP^OX^*. After homozygous plants for both transgenes were obtained, double transgenic lines expressing *GFP* to the same levels as the *GFP^OX^* line in the Col-0 background were selected and used for analyses.

### 4.2. Transient Protoplast Expression Assay

Protoplast isolation and polyethylene glycol-mediated transfection were performed as previously described [28]. The effector constructs that were overexpressing *OsSnRK1A^WT^* or *AtSnRK1.1^WT^* were generated previously [20,21]. To generate reporters for translated proteins, the full-length coding sequences of *GFP* and *SPYNE* were amplified and cloned downstream of the *35SC4PPDK* promoter (the cauliflower mosaic virus [CaMV] 35S enhancer fused to the maize *C4PPDK* basal promoter) and upstream of firefly luciferase (*fLUC*) in pHBT-fLUC. After a 6-h incubation in WI solution, the protoplasts were harvested and analyzed. The reporter activities were measured with a luciferase system (Promega). Renilla luciferase activity (rLUC) was used as an internal control to normalize fLUC activity. All experiments were performed at least three times with similar results.

### 4.3. Immunoblot Analysis

Total proteins were extracted from plants or protoplasts using extraction buffer (50 mM Tris-Base, 150 mM NaCl, 10 mM NaF, 10 mM Na_3_Vo_4_, 1x protease inhibitor cocktail, and 0.2% [*v/v*] Triton X-100). After the cell lysate was centrifuged, the total proteins in the supernatants were separated by SDS-PAGE and transferred to polyvinylidene difluoride membranes. For immunoblotting, the primary antibodies anti-GFP (Abcam, Cambridge, UK); anti-pT172-AMPKα antibody (Cell Signaling Technology, Danvers, MA, USA); and anti-Actin (Agrisera, Vännäs, Sweden) were used (1:1000), and then an HRP-conjugated secondary antibody (Abcam, Cambridge, UK) was added (1:10,000). The signal was detected using a Fusion SL (Vilber Lourmat, Paris, France). All experiments were performed at least three times with similar results. Representative protein blot data are shown.

### 4.4. RNA Extraction and Gene Expression Analysis

Total RNA extraction and first-strand cDNA synthesis were performed as previously described [29]. qPCR was performed using gene-specific primers (Table S1) and conducted on a QuantStudio 3 Real-Time PCR System (Thermo Fisher Scientific, Waltham, MA, USA) using the SYBR Green Master Mix (Bio-Rad, Hercules, CA, USA). Gene expression was quantified using the comparative Ct method. *AtACTIN* or *DAP1* were used as internal controls. All experiments were performed at least three times with similar results.

### 4.5. Cell Free Degradation Assay

For the cell-free degradation assay, the pHBT-GFP vector was transfected into protoplasts. After an 8-h incubation in W5 solution, the GFP protein was purified using a GFP-Trap (ChromoTek, Planegg, Germany) as per the manufacturer’s instructions. Cell-free degradation assays were performed as previously described [30]. Briefly, frozen seedling powders of Col-0, *OsSnRK1A^W^^T^*, and *AtSnRK1.1^W^^T^* were homogenized and ground with the degradation assay buffer containing 25 mM Tris-HCl [pH 7.5], 10 mM NaCl, 10 mM MgCl_2_, 5 mM DTT, 10 mM ATP, and 4 mM PMSF. After centrifugation, the supernatants were adjusted to equal concentration with the degradation assay buffer and then incubated with the purified GFP proteins for the indicated times. The reaction was stopped by 5× SDS-PAGE sample buffer and then the immunoblots were performed with anti-GFP (Abcam, Cambridge, UK). All experiments were performed at least three times with similar results. Representative protein blot data are shown.

### 4.6. Analysis of Polysome-Bound RNA Abundance

Analysis of the polysome-bound mRNAs was performed as previously described [17,31]. Briefly, frozen seedlings were ground to powder and resuspended in polysome extraction buffer containing 200 mM Tris-HCl pH 8.0; 50 mM KCl; 25 mM MgCl_2_; 50 ug mL^–1^ cycloheximide; 400 U mL^–1^ RNasin with 1:500 (*v/v*) protease inhibitor cocktail; 2% (*w/v*) polyoxyethylene-10-tridecyl ether; and 1% (*w/v*) sodium deoxycholate. After centrifugation, the supernatant was separated on a 10-mL continuous sucrose gradient (15–50%, *w/v*), after which the RNA distribution was determined based on absorbance under an ultraviolet (UV) light at 254 nm. The RNAs were separated into NP, LP, and HP fractions using a pipette with the total amount of mRNA in the three fractions set to 100%. The percentage of total mRNA was determined as follows: The amount of mRNA in each fraction/amount of total mRNA. The amount of *GFP* mRNA in polysome fractions was measured by RT-qPCR with *DAP1* as an internal reference. The results were quantified as a percentage of the total amount in the three fractions.

### 4.7. Statistical Analysis

All experiments were performed at least three times and the data were analyzed by a *t*-test using GraphPad Prism 8.0 software. The asterisks indicate significantly different values (* *p* < 0.05 and ** *p* < 0.01).

## Data Availability

The data presented in this study are available in the article or the Supplementary Materials.

## Supplementary Materials

The following supporting information can be downloaded at: https://www.mdpi.com/article/10.3390/plants11101359/s1, Table S1: Sequence of primers used in this study.
