# Supplementary material for: SNF1-Related Protein Kinase 1 Activity Represses the Canonical Translational Machinery
Source: Plants (Basel). 2022 May 20;11(10):1359. doi: 10.3390/plants11101359 (PMC9147276; doi:10.3390/plants11101359)
Supplement: Supplementary file 1 [file plants-11-01359-s001.zip › plants-1706052-supplementary.pdf]

Gene	Primer sequence	Purpose
GFP	F: TTCTTCAAGGACGACGGCAA	qRT-PCR
	R: TCGATGTTGTGGCGGATCTT	qRT-PCR
SPYNE	F: GTGACCACCTTCGGCTACGG	qRT-PCR
	R: CCCTTCAGCTCGATGCGGTT	qRT-PCR
AtACTIN	F: ATGTGGATCTCCAAGGCCGA	qRT-PCR
	R: ACACACAAGTGCATCATAGAAACGAA	qRT-PCR
DAP	F: CAGACCGCGGCCTAATAATG	qRT-PCR
	R: CAGTGACAATTGACCGAATTGC	qRT-PCR

Table S1. Sequence of primers used in this study
